# Clinical applications of exosome membrane proteins

**DOI:** 10.1093/pcmedi/pbaa007

**Published:** 2020-02-24

**Authors:** Qian Hu, Hang Su, Juan Li, Christopher Lyon, Wenfu Tang, Meihua Wan, Tony Ye Hu

**Affiliations:** 1 Center of Cellular and Molecular Diagnosis, Tulane University School of Medicine, New Orleans, LA 70112, USA; 2 Department of Integrated Traditional Chinese and Western Medicine, West China Hospital of Sichuan University, Chengdu 610041, Sichuan Province, China; 3 Health Management Center, West China Hospital of Sichuan University, Chengdu 610041, Sichuan Province, China; 4 Department of Biochemistry & Molecular Biology, Tulane University School of Medicine, New Orleans, LA 70112, USA

**Keywords:** Exosome, membrane protein, endocytosis, membrane fusion, diagnostic markers, exosome mimetics, target therapy

## Abstract

Extracellular vesicles (EVs) are small membranous particles that can mediate cell-to-cell communication and which are divided into at least three categories according to their subcellular origin and size: exosomes, microvesicles, and apoptotic bodies. Exosomes are the smallest (30–150 nm) of these EVs, and play an important role in EV-mediated cell-to-cell interactions, by transferring proteins, nucleic acids and, lipids from their parental cells to adjacent or distant cells to alter their phenotypes. Most exosome studies in the past two decades have focused on their nucleic acid composition and their transfer of mRNAs and microRNAs to neighboring cells. However, exosomes also carry specific membrane proteins that can identify the physiological and pathological states of their parental cells or indicate their preferential target cells or tissues. Exosome membrane protein expression can also be directly employed or modified to allow exosomes to serve as drug delivery systems and therapeutic platforms, including in targeted therapy approaches. This review will briefly summarize information on exosome membrane proteins components and their role in exosome–cell interactions, including proteins associated with specific cell-interactions and diseases, and the potential for using exosome membrane proteins in therapeutic targeting approaches.

## Introduction

Exosomes derive from the inward budding of the late endosomal membranes in a process that generates multi-vesicular endosomes (MVEs) that subsequently fuse with the plasma membrane to release exosomes into the extracellular space.^1^ These vesicles provide a broad array of biological and genetic information that can reflect the phenotype of the parental cell (microRNAs, mRNAs, long non-coding RNAs, DNA fragments and proteins) and alter the phenotype of recipient cells that take up these vesicles.^2^^,^^3^ Because of their small size and relative durability, exosomes readily transit from their site of origin during tissue-specific secretion processes or tissue perfusion to accumulate in serum and most other bodily fluids, including cerebrospinal fluid, saliva, and urine. Exosomes secreted by a given parental cell, including diseased cells, thus have the potential to affect the behavior of adjacent cells, the microenvironment of their parental cell, and the phenotype of distant cells and tissues, with the potential to produce systemic effects. As exosomes can also be detected in most body fluids, analysis of exosomes present in samples obtained by non-invasive or minimally invasive methods has the potential to detect pathological changes that would otherwise require a tissue biopsy, which may not always be feasible. Exosomes are thus of great interest as disease biomarkers, although more work needs to be done to identify and validate the diagnostic ability of exosome-associated biomarkers for specific diseases. The innate properties of exosomes, including their stability and potential tissue or cell selectivity also make them good candidates for therapeutic approaches, as do their low immunogenicity and ideal biocompatibility, which are better than those of microvesicles and apoptotic bodies.^3^ However, the mechanisms through which exosomes recognize specific target cells to regulate their behavior are not well understood and remain a subject of great interest.

Exosomes from different cell types often carry distinct RNA and protein cargoes that reflect the phenotypes of their parental cells,^4^ and can carry cell-specific or tissue-specific factors that can be used to identify their site of origin. Exosomes secreted from any given cell type can also exhibit divergent cargo profiles when subjected to different environmental conditions and stresses, such as those encountered during pathological states, including cancer and chronic and infectious disease. Multiple studies have focused on the roles of exosome RNA and DNA cargoes in intercellular signaling and pathological responses,^2^^,^^3^ but mounting evidence indicates that exosome membrane proteins also play important roles in these events, and have emerged as promising diagnostic biomarkers and therapeutic targets.^4–7^

Exosomes released by healthy cells exhibit membrane protein expression profiles distinct from those of matching cells that have undergone differentiation events, malignant transformation, or which have been infected with a microbial pathogen.^5–7^ Several studies have now indicated that multiple exosome membrane proteins play key roles in these processes, such as promoting tumor invasion and metastasis,^8^^,^^9^ inhibiting immune responses,^10^ or expanding the range of cells accessible for viral or bacterial infection.^11^^,^^12^

Discovery of the innate and modifiable regulatory activities of exosomes has led to substantial research aimed at directly modifying or engineering exosomes to function as carriers of therapeutic drugs. Selective surface modification of exosomes with targeting proteins or peptides and/or therapeutic drugs is a major strategy for exosome-based therapeutics.^13^ Several such methods in current use (surface loading of native exosomes, genetic modification of their parental cells, and generating exosome mimetics by coating nanoparticles with exosome membrane material) have advantages and disadvantages. These approaches demonstrate potential for clinical applications, although technical and unforeseen side effects may limit the future utility of some exosome-based therapeutic approaches.

This review focuses on the role of membrane proteins in potential exosome therapeutics, and will discuss select exosome membrane proteins and their role in exosome-mediated cell communication, including proteins associated with cell- or tissue-specific exosome interactions, changes in these protein under pathological conditions, and the application of specific membrane proteins in disease diagnosis and treatment. Finally, this review will discuss the potential advantages and disadvantages of applications that employ, modify, or mimic exosome properties for clinical therapeutics.

## Exosome membrane composition

Exosomes can carry a multitude of diverse factors, and more research is required to evaluate what roles specific factors play in different physiological and pathological process. Exosomes contain an array of membrane-associated lipids and proteins in addition to their lipid, protein coding and non-coding RNA cargoes.^6^ Most studies analyzing changes in exosome composition associated with specific pathologies have focused on characterizing functional changes in exosome RNA cargoes, but there is growing interest in the potential regulatory roles of exosome membrane proteins. The exosome membrane is generated by two sequential membrane invaginations, an inward budding of the plasma membrane first gives rise to the late endosomal compartment, after which a second regulated membrane invagination at the endosomal outer membrane serves to selectively package cytosolic and membrane components into vesicles that bud into the endosomal lumen.^6^ This biogenesis process involves the regulated sorting of components from multiple distinct membranes with different functions and compositions for incorporation into the exosome membrane, including lipids and proteins associated with the membranes of the Golgi apparatus, the endoplasmic reticulum, and the plasma membrane. Exosome membranes contain plasma membrane proteins, including ligands and receptors that can promote their interaction with their target cells, and which can confer some degree of cell-specificity. This membrane composition also contributes to the low immunogenicity of exosomes upon their exposure to the systemic immune repertoire.^14^ However, exosomes are also enriched in a subset of factors during their biogenesis so that their compositions can significantly diverge from those of their parental cells,^3^ altering exosome membrane composition and functionality and allowing their discrimination from other circulating extracellular vesicle populations. For example, exosomes from a variety of different cell types exhibit enrichment in cholesterol, sphingomyelin, and hexosylceramides and reductions in phosphatidylcholine and phosphatidylethanolamine, relative to the plasma membranes of their parental cells.^15^

Many exosome-specific or -selective proteins are displayed on exosome membranes, including several tetraspanin proteins (e.g. CD81, CD82, CD37, and CD63) and proteins involved in cell adhesion and signaling, cytoskeletal structures, lipid rafts, and membrane trafficking.^16^ These include two members of the endosomal sorting complex required for transport (ESCORT) pathway, Alix and TSG101, which are frequently used as exosome markers because of their central role in exosome cargo sorting.^17^ Several exosome-specific membrane proteins, such as Alix, TSG101, and Rab5 and multiple tetraspanins, are employed to distinguish exosome from microvesicles and apoptotic bodies by immunological methods (e.g. western blots, immunohistochemistry analyses, and ELISAs). Commercial assays employing antibodies specific for conserved exosome membrane proteins have also been used to isolate exosomes directly from cell culture supernatants and biological samples.^18^

Despite the prevalence of a select set of proteins on exosomes derived from diverse cell types, exosomes can exhibit a large array of proteins depending on their parental cell type, with the Exocarta database (http://www.exocarta.org) listing 41 860 exosome-associated proteins identified among 10 analyzed species.

## Exosome membrane proteins in intercellular communication

**Figure 1:**
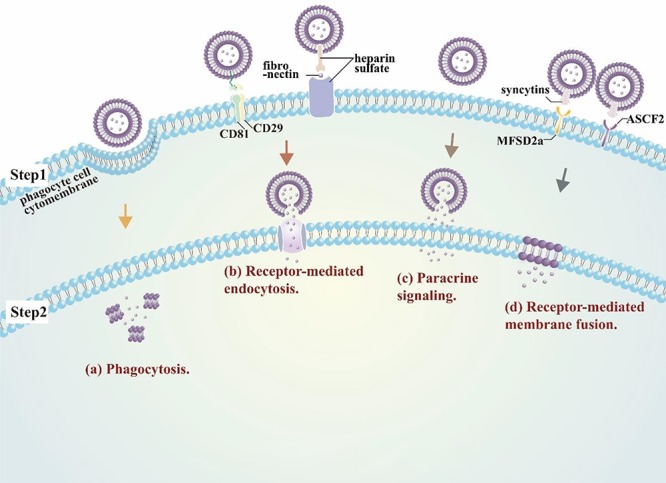
**Mechanism of EV to cell interactions.** Exosome uptake appears primarily mediated by (**a**) the standard phagocytosis machinery in professional phagocytes, and (**b**) receptor-mediated endocytosis in other cell types (e.g. CD29/CD81- or fibronectin-mediated interactions). However, exosomes also exhibit **(c)** paracrine signaling, where factors released by exosomes can directly adhere to cell surface receptors, and **(d)** receptor-mediated membrane fusion *via* interactions between exosome and cell membrane proteins (e.g. syncytins and MFSD2a or ASCF2).

Exosomes can regulate the behavior of their target cells through direct or indirect delivery of their cargoes to these recipient cells. There are at least four different reported mechanisms responsible for exosome–cell interactions (Fig. 1) that can regulate cell behavior: receptor-mediated exosome uptake, protein-mediated fusion of the exosome and plasma membranes, phagocytosis, and a paracrine signaling process that can arise from spontaneous release of exosome cargoes upon the destabilization of exosome membranes under low pH conditions.^17^^,^^19^^,^^20^ In the paracrine mechanism, factors released by the exosomes directly adhere to the surface of the recipient cells through factor-specific mechanisms to separately exert their regulatory effects,^19^ while in all other cases exosome effects appear to be primarily regulated by interactions between factors on the surface of an exosome and its recipient cell that result in a coordinated transfer of exosome cargoes. Better understanding of the factors and mechanisms that govern general and cell-selective exosome cargo transfer is of great importance in developing improved exosome-based therapeutics.

### Receptor-mediated endocytosis

Endocytosis is a fundamental cellular process that uses an ancient, evolutionarily conserved network of proteins to internalize nutrients and maintain cellular homeostasis,^21^ and may represent the primary means of exosome uptake. Endocytosis can occur through at least four distinct uptake pathways, including caveolae-dependent and clathrin-dependent endocytosis, micropinocytosis, and phagocytosis, all of which are reported to regulate exosome uptake.^22^

Exosome uptake by professional phagocytes, such as macrophages and dendritic cells, appears to be primarily regulated by phagocytosis, as this process can be markedly attenuated by inhibiting phagocytosis via dynamin 2 knockdown or treatment with the specific inhibitor latrunculin-A.^19^^,^^23^ Multiple different mechanisms have been reported to influence exosome uptake in other cell types, where exosomes are reported to adhere to the cell surface through protein–protein or receptor–ligand interactions to initiate signaling cascades that activate different endocytosis pathways.^22^ One report indicates that a fibronectin-mediated linkage of heparin sulfate on the surface of exosomes and target cells plays an important role in exosome–cell interactions not mediated by phagocytosis.^24^ In this study, fibronectin bound to exosomes isolated from myeloma cell cultures was found to regulate exosome–cell interaction, degradation of heparin sulfate on the surface of the exosomes or their recipient cells was found to attenuate this interaction, and this interaction could be blocked by heparin sulfate mimetics or antibody blockade of the heparin-II domain of fibronectin. Notably, this study included the use of a heparin sulfate mimetic, roneparstat, which has been reported to inhibit the growth of myeloma tumors in mouse models, albeit via a different proposed mechanism,^25^ and reported to be safe and well-tolerated in a phase I clinical trial, although evaluation of its treatment efficacy was beyond the scope of this study.^26^ Results from other studies suggest that exosomes derived from non-malignant cell populations may also use a fibronectin-heparin sulfate linkage mechanism to interact with their recipient as since fibronectin is abundant in the circulation and on cell surfaces, exosomes can be isolated from the plasma of normal subjects using heparin affinity beads, and heparin incubation or treatment with heparan sulfate-degrading enzymes can attenuate exosome–cell interactions.^27–29^

It has also been reported that an integrin–tetraspanin complex can regulate exosome uptake, as one study has reported that radiation treatment of exosome recipient cells can increase their exosome uptake via a process that increases co-localization of CD29 and CD81 on the recipient cells, without altering the expression of either of these proteins, and without affecting the expression or distribution of any other assayed tetraspanin protein (CD9, CD63, and CD151).^30^ This study reported that CD29 knockdown completely inhibited radiation-induced exosome uptake and that CD81 knockdown inhibited both basal and radiation-induced exosome uptake, but did not identify the exosome membrane factor that associated with this complex.

As endocytosis appears to be primarily responsible for exosome uptake, which is required for most exosome-mediated effects to alter the phenotype of their recipient cells, several approaches using well-known inhibitors of endocytosis have been examined for their ability to block exosome uptake and their regulatory effects, including shRNA transfection, loss of function mutations, and small molecular inhibitors, such as genistein and nystatin.^22^^,^^31^ Such broad-spectrum approaches are not feasible for *in vivo* therapeutic interventions, but it may be possible to inhibit interactions between specific exosome and cell populations by blocking receptors involved in these selective events.

### Receptor-mediated membrane fusion

While endocytosis appears to represent the dominant means by which exosomes interact with and influence the phenotype of their recipient cells, exosome-associated proteins have also been implicated in directly regulating the fusion of the exosome and plasma membranes.^32^ For example, Syncytin-1 and -2 have been implicated in the cell fusion events of placenta-derived exosomes and have high affinity for two transmembrane proteins, lipid symporter MFSD2A and the neutral amino transporter ASCT2, which exhibit broad tissue expression and may serve as a general, non-selective means of exosome fusion with the plasma membranes of their recipient cells.^14^ However, while interactions between syncytins and MFSD2A and ASCT2 may serve to initiate membrane fusions between exosomes and their target cells, completion of the process requires the activity of additional factors involved in protein restructuring, membrane dimpling, and lipid reorganization.

Receptor-mediated membrane fusion has also been implicated in another reproductive process, the interaction of eggs and sperm. EVs are released from the perivitelline space of mammalian eggs just prior to fertilization in a mechanism that appears to facilitate fusion of the sperm and egg cell membranes via a process that requires expression of IZUMO1 by sperm and CD9 by the egg.^33^^,^^34^

## Lineage-specific and disease-specific exosome membrane proteins

Exosome membrane compositions can vary based on cell origin, as well as the physiological state of the parent cell during exosome biogenesis, including changes associated with chronic and infectious diseases, suggesting that analysis of specific circulating exosome populations could provide valuable information about the physiologic state of tissues that would be otherwise difficult to evaluate because of their inaccessibility or the need to sample multiple discrete sites.

### Lineage-specific exosome proteins

As surface-bound proteins on exosomes are influenced by the repertoire of proteins expressed on the plasma membranes of their parental cells, exosome membrane compositions can vary based on both the type and the physiological state of their parental cells.

The circulating exosome population is highly diverse, as it reflects the aggregate contributions of all cells and tissues collected during normal systemic perfusion. The ability to detect, isolate, and analyze tissue-specific or cell-specific exosomes in this mixed population could provide valuable information about the physiologic state of tissues that would be otherwise difficult to evaluate because of their inaccessibility or the need to sample multiple discrete sites. The potential of such approaches is limited by the lack of validated cell-specific biomarkers, in part resulting from the technical challenges associated with identifying such markers.^35^

Studies designed to analyze cell-specific exosome biomarkers typically employ proteomics to analyze the differential composition of exosomes derived from distinct cell or tissue sources, and results can be influenced by the state of the cells or tissue analyzed in the study and the purity of the source cells and resulting exosome preparations. Research is ongoing to identify and validate markers that can distinguish exosomes derived from infected cells or that can distinguish or target specific cell types, because of the great potential for diagnostic and therapeutic applications that could be developed on validation of such markers. For example, one recent proteomics study performed with exosomes isolated from primary rat hepatocyte cultures has proposed that the transmembrane protein ASGR represents a specific marker for hepatocyte-derived exosomes,^36^ but whether ASGR is also a specific protein of human hepatogenic cells requires further investigation. Multiple studies have now identified factors that can function as biomarkers for exosomes derived from specific cells, cell lineages, or tissues (Table 1), but further studies are required to validate the specificity of these biomarkers for their target exosome populations.

**Table 1:** Function of cell-specific and disease-specific membrane proteins of exosomes

**Parent cell type**	**Protein biomarker**	**Function or utility**	**References**
Hepatocyte cells	ASGR	Identify hepatocyte-derived exosomes	36
B cell	Major histocompatibility complex (MHC) class II	Stimulate CD4^+^ T cell response	37,38
*M. avium*-infected macrophages	*M. avium* glycopeptidolipids	Toll-like receptor ligands	39
Mature dendritic cell	MHC class II, Intercellular adhesion molecule 1 (ICAM1)	Activate T cell responses	40
Non-small-cell lung cancer cell	Lipopolysaccharide binding proteins (LBP)	Identify metastatic NSCLC tumors	45
Ovarian cancer cell	Soluble E-cadherin	Identify metastatic ovarian cancer	8
Metastatic cancer cell	Integrins α6β4, α6β1 and αvβ5	Predict organ-specific metastasis	9
Metastatic melanomas	PD-L1	Identify tumors non-responsive to chemotherapy	10
Circulating exosomes	Collagens, vimentin and fibronectin	Identify stable transplant phenotypes	46

Antigen-presenting cells (APCs), including dendritic cells (DCs), macrophages and B cells, secrete exosomes that tend to display a surface pattern of immune regulatory proteins and antigens similar to that of their parental cells. Exosome display or delivery of these proteins can exert the same effects as their expression on their parent cells, stimulating CD4^+^ T cell responses,^37^^,^^38^ activating pro-inflammatory response,^11^^,^^12^ or priming protective immune responses to prevent infection.^39^ Many of these proteins may serve as potential candidates for biomarkers of the lineage-specific or cell-type-specific origin of target exosome populations.

These proteins can be directly exploited for exosome therapeutics. For example, exosomes isolated from macrophages treated with *M. tuberculosis* culture filtrate protein, have been employed to develop an exosome-based vaccine which has the ability to activate an immune response to this pathogen.^39^ A phase II clinical trial administering dendritic cell-derived exosomes to patients with advanced non-small cell lung cancer (NSCLC) found that these exosomes could be used as maintenance immunotherapy after induction chemotherapy without tumor progression, and boosted the natural killer cell aspect of antitumor immunity in these patients.^40^ Conversely, it is also necessary to account for these properties when selecting exosomes for research and clinical applications, where it is best to select exosomes matching the host to maintain their low immunogenicity and high compatibility. For example, exosomes released by autologous APCs are safer to use in patients with NSCLC, based on the evidence of phase I study on the long-term stability of disease and activation of immune effectors in exosome-treated NSCLC patients.^41^

### Disease-specific exosome proteins

Exosomes produced by cells experiencing pathological conditions or other stresses can exhibit altered compositions to serve as potential biomarkers of these states. Specific stressors known to alter exosome composition include specific cellular dysfunctions, cancers, and infections.

Exosome expression of pathogen-derived factors represents a clear case where the detection of a target biomarker on a circulating exosome population represents strong evidence of the linked disease. Exosome-derived biomarkers for chronic non-infectious diseases and conditions are more challenging to use as they often reflect altered expression of a protein that may already be expressed at significant levels in the general exosome populations, and thus may require target exosomes to be isolated for analysis or that a threshold be employed to discriminate expression levels characteristic of pathologic versus non-pathologic conditions.^42^^,^^43^ Nonetheless, multiple studies have identified exosome proteins that are associated with cancer, metastasis and other non-malignant pathological conditions.^8^^,^^11^^,^^12^^,^^36–38^^,^^44^^,^^45^

**Figure 2:**
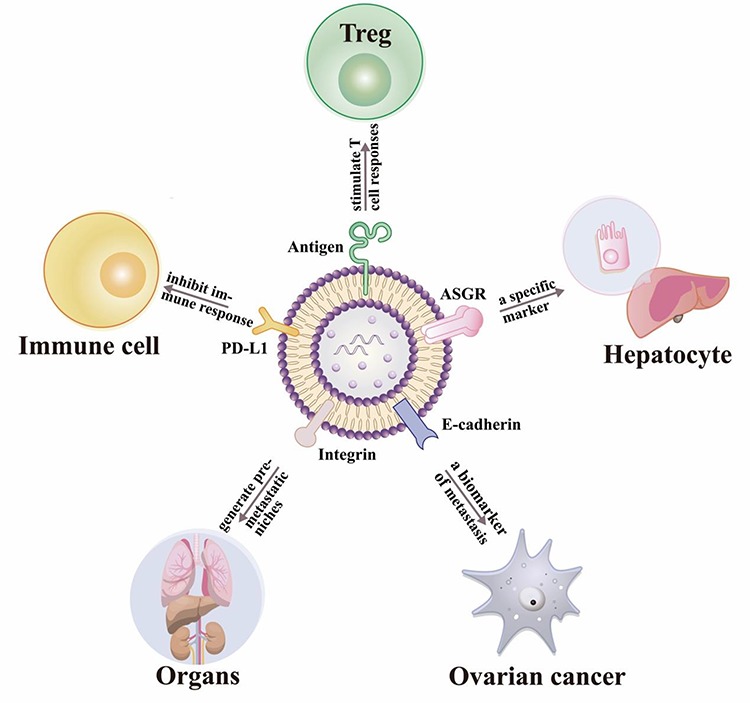
**The special functions of some exosome membrane proteins.** 1) **Immune cell activation** (e.g. antigen-presenting cells secrete exosomes that carry MHC II-antigen complexes that can stimulate T-cell responses); 2) **Cell-specific markers** (e.g. the transmembrane protein ASGR can serve as a specific marker for exosomes derived from hepatocytes); 3) **Metastatic potential** (e.g. exosomes expressing E-cadherin which can promote ovarian cancer metastasis); 4) **Exosome homing** (e.g. integrins on cancer-derived exosomes can predict organs at a risk for future metastasis); 5) **Immune cell repression** (e.g. exosome PD-L1 expression can indirectly inhibit immune responses).

Exosome membrane compositions can change during disease progression, and may thus be useful as diagnostic or predictive biomarkers of the current disease stage and the potential for progression to more severe or advanced disease. Exosomes derived from metastatic cells are reported to carry factors that promote cancer cell proliferation, migration, invasion and angiogenesis, while exosomes from non-metastatic cells tend to contain proteins involved in cell–cell or cell–matrix adhesion and polarity maintenance.^44^ This phenomenon has been observed for a variety of different cancers, including breast, colorectal, and non-small-cell lung cancers.^4^^,^^44^^,^^45^ Metastatic ovarian cancer cells abundantly secrete exosomes that express soluble E-cadherin, an angiogenesis inducer, and heterodimerize with VE-cadherin expressed on endothelial cells to activate β-catenin and NFКB signaling, suggesting that increased expression of soluble E-cadherin on exosomes of ovarian origin could serve as a diagnostic and prognostic biomarker for ovarian cancer.^8^ Exosome expression of lipopolysaccharide-binding proteins (LBP) and soluble E-cadherin have also been used to distinguish NSCLC and ovarian cancer cells with metastatic and non-metastatic phenotypes.^8^^,^^45^ Integrin expression on cancer-derived exosomes can also predict tissues at risk for future metastasis, with exosome expression of integrin α6β4 and α6β1 associated with lung metastasis, and integrin αvβ5 expression linked to liver metastasis.^9^ Metastatic melanoma secretion of exosomes that express programmed death ligand-1 (PD-L1), which can interact with the programmed death-1 (PD-1) receptor on T cells to initiate the immune checkpoint response, can also serve as an indicator of the adaptive response of the tumor cells to T cell reinvigoration.^10^Figure 2 illustrates select exosome membrane proteins and their reported functions.

Circulating levels of cell- or tissue-restricted exosome proteins (Table 1) can also be used to diagnose pathological conditions associated with chronic rejection reactions following organ transplantation, as individuals with chronic rejection versus stable phenotypes were found to demonstrate significantly elevated exosome expression of tissue-restricted factors.^46^ Circulating exosomes have demonstrated increased expression of collagen V and K-α 1 tubulin in lung transplant recipients with bronchiolitis obliterans syndrome; cardiac myosin and vimentin expression in individuals with coronary artery vasculopathy following heart transplant; and collagen-IV and fibronectin in kidney transplant recipients with transplant glomerulopathy.^46^ Notably, protein expression differences between individuals with chronic rejection and stable transplant phenotypes were relatively robust, ranging from greater than 2-fold in kidney transplant patients, to approximately 4-fold or greater in lung and heart transplant recipients, implying that such exosomes expressing such tissue-selective proteins have the potential to function as noninvasive biomarkers for allograft rejection.

## Exosome membrane protein therapeutics

Exosomes have multiple advantages for the delivery of therapeutic cargos.^47^ They exhibit low immunogenicity, are stable under physiological conditions, and can cross the blood-brain barrier. The highly stable nature of the exosome lipid bilayer serves to protect their cargoes from the immune system and circulating hydrolases, while specific membrane proteins can facilitate the delivery of their contents to targeted recipient cells by endocytosis or membrane fusion events to preserve the intrinsic function of these cargoes during transfer. Exosome membranes can be modified for a wide range of applications. For example, peptides that induce exosomes to home to diseased tissues have been loaded on exosome surfaces to direct drug accumulation at a target site.^13^^,^^48^ By October 2019, there were 54 exosome-related clinical trials listed at clinicaltrials.gov when “exosome” was used as a search term, indicating the growing interest in exosomes for clinical applications. The sections below briefly summarize the major exosome-related therapeutic approaches under development.

### Non-modified exosomes as therapeutic agents

Exosome membranes are enriched in tetraspanin and heat shock proteins when compared to those of their parental cells, and these factors and other factors present on membranes of exosomes derived from healthy cells can exert beneficial physiologic effects on their recipient cells.^16^^,^^33^^,^^34^ Exosomes isolated from healthy cells thus have the potential to serve as natural therapeutic agents. Such exosomes would avoid the potential for unforeseen side effects that could arise when using exosomes that have been engineered to carry specific targeting or therapeutic modifications.

Exosome proteins that exhibit broad and restricted expression profiles have both been reported to exhibit specific therapeutic potential. For example, in the former group, exosome CD9 expression has been linked to osteoclastogenesis that can promote osteoblast fusion and bone healing,^34^ and expression of heat shock proteins on exosome membranes is reported to have cardio-protective effects in models of cardiac ischemia-reperfusion injury, by attenuating TLR4 signaling and stimulating inflammatory cytokine release.^49^^,^^50^ Similarly, exosome expression of secretory carrier membrane protein 5 (SCAMP5) is reported to colocalize with and mediate clearance of α-synuclein to attenuate α-synuclein aggregation in neurodegenerative diseases and rescue neuronal function impairment and cell death.^51^ Several studies now indicate that membrane proteins on exosomes secreted by healthy cells can regulate tissue homoeostasis by attenuating injury responses, and promoting clearance and repair processes. Such exosome therapeutics should be relatively safe to administer, but the development of such approaches is limited by the lack of knowledge regarding the functional effects of specific membrane proteins present on exosomes, and technical issues Ewith exosome purification and scaling exosome production, which have slowed progress towards clinical trials of natural exosome therapeutics.

### Non-recombinant exosome modifications for targeted delivery of therapeutics

Most clinically approved drugs are rapidly cleared from the circulation and non-specifically distributed through body tissues so that only a small fraction of the administered dose reaches its intended target site, leading to low efficacy and adverse side effects.^52^ Exosomes are attractive nanocarriers for the targeted delivery of therapeutics, because of their stability, biocompatibility, low immunogenicity and toxicity, and efficient cellular uptake that can be targeted to specific cells and tissues by their surface display of specific proteins and ligands. However, there is a limited degree of target specificity that can be achieved using native exosomes with inherent tissue cell or tissue selectivity, and after administration such exosomes tend to accumulate in liver, kidney, and spleen, where they are rapidly cleared by bile excretion, renal filtration, or reticuloendothelial system phagocytosis, respectively.^20^ Therefore, exosome targeting approaches that can increase the range of targeted tissues and reduce the fraction of therapeutic exosomes lost to non-specific clearance mechanisms are of great interest.

Several approaches can be employed to directly modify the surface of native exosomes to display ligands or receptors and therapeutic agents facilitate efficient high concentration delivery of therapeutic drugs to specific target sites. Relatively simple chemical and physical methods can be employed to add therapeutic drugs to the exosome surface, or its cargo, to expand the range of functional targets and applications that can be addressed by exosome membrane-based therapy. One approach is to use a freeze–thaw process to generate exosome–liposome hybrids that exhibit the targeting properties of exosomes and the surface and cargo modification potential of liposomes, to transfer both hydrophobic and hydrophilic therapeutic agents.^53^^,^^54^

Mixing exosomes directly with candidate proteins to modify their specificity or alter the ability to induce functional changes in their recipient cells has not proven successful, however, as this approach has resulted in large, unstable and/or aggregated exosomes, as well as undesired chemical contamination. Such surface display approaches are also vulnerable to microenvironment changes encountered during administration that could cause adhered proteins to dissociate, resulting in loss of the target specificity or functionality. Exosomes modified by such surface display approaches have not been used for clinical applications because of concerns about their stability and safety.

Non–covalent exosome modification approaches have yet to prove viable for exosome targeting or therapeutic delivery, but chemical conjugation has been successfully employed to confer target specificity on native exosomes. One such approach used a reactive crosslinker approach to attach reactive dibenzolcyclooctyne groups onto amine-containing molecules on native exosomes, and subsequently reacted these groups with a cyclo (Arg-Gly-Asp-D-Tyr-Lys) [c(RGDyK)] peptide that exhibits specificity for integrin αvβ3, which is expressed on cerebral vascular endothelial in response to ischemia.^48^ Notably, c(RGDyK)-modified exosomes loaded with curcumin were found to suppress inflammation and cellular apoptosis in a transient middle cerebral artery occlusion mouse model after intravenous injection, demonstrating that these modified exosomes were able to transit the blood–brain barrier to exert their therapeutic effect.^48^ A more recent study used phage display to identify an anchor peptide (CP05) that binds CD63 with high affinity and specificity to allow non–covalent CD63-mediated modification of EVs.^37^ In this approach, isolated exosomes are incubated with a cell- or tissue-specific molecule tagged with CP05 to alter their target specificity, a CP05-tagged therapeutic agent to alter therapeutic potential, or both to enable targeted delivery of therapeutic exosomes to a target tissue. This modular targeting approach avoids the need to identify and highly purify exosomes with a desired target specificity for therapeutic delivery or to engineer cells lines that produce exosomes with a desired target specificity.

Such direct modification approaches to alter exosome specificity have the advantage that they do not require the identification and rigorous purification of exosomes with a desired target specificity. However, covalent exosome modification approaches carry the risk of undesirable chemical contamination or modification of the altered targeting exosomes, while non-covalent modification approaches may produce exosomes with unstable target specificity under physiological conditions. The efficiency of exosome modification may also be an issue for both covalent and noncovalent approaches, resulting in exosome populations with variable purity and raising the potential for off-target effects.

### Recombinant exosome modifications for targeted delivery of therapeutics

Recombinant DNA approaches have also been employed to generate cell lines that secrete exosomes with desired target specificities, avoiding potential chemical toxicity and receptor instability problems possible with chemically or non-covalently modified exosomes, while ensuring that all exosomes have the desired target specificity. Several groups have used recombinant DNA approaches to produce exosomes with engineered therapeutic or targeting potentials. For example, genetic engineering has been used to modify HEK293T cells to generate exosomes that overexpress SIRPα to act as a cancer therapeutic by disrupting CD47-SIRPα interactions between tumor cells and macrophages to attenuate the ability of tumor cells to resist phagocytosis.^55^ Engineered exosomes have also been used in cancer vaccine approaches, where exosomes derived from murine embryonic stem cells engineered to produce GM-CSF were found to attenuate tumor development in a mouse model of lung cancer.^56^ Genetic modification has also been used to modify HEK293 cells to secrete exosomes that express a peptide (GE11) that specifically binds to EGFR, promoting their interaction with EGFR-positive breast cancer cells and their delivery of a therapeutic miRNA (let-7a) to attenuate tumor development in a mouse model of breast cancer.^57^

Modifying exosome membrane expression by genetic alterations to parental cells should produce more uniform exosome populations, with potentially more stable targeting specificities, than other methods. However, genetic modification approaches are also more time-consuming and expensive than direct modification approaches to alter target specificity, and can raise potential safety concerns that may complicate translation to clinical applications.

### Membrane-coated exosome mimetics

Neither the surface display nor the genetic engineering approaches described above address the issue of exosome purification, which can require large sample volumes to obtain sufficient material for therapeutic applications. Exosome mimetics, nanoparticles coated with membranes that simulate those of exosomes with desired targeting or functional characteristics, have become a promising means to address low yields associated with purification of endogenous endosomes for therapeutic applications. Exosome mimetics can be rapidly and cost-effectively engineered to carry the functional and targeting properties of exosomes coupled with the drug loading properties of their nanoparticle cores, and have recently received considerable attention as effective drug delivery platforms. Membrane-coated nanoparticles generated by extruding biocompatible nanoparticles through a nanoporous membrane in the presence of membrane isolates from cells with desired functional properties reveal size distribution, morphology, stability and immunocompatibility characteristics similar to the characteristics of exosomes produced by these cells.^58^ Notably, this production process is highly controllable, and can be engineered to reproducibly produce high yields of pharmaceutical grade exosome mimetics, with high loading capacities, which are suitable for use in preclinical or clinical settings.^59^

Tumor-derived exosome membranes can carry both tumor antigens and exhibit specific cell-homing properties. Nanoparticles coated with cancer cell membranes (CCNPs) can promote tumor-specific immune responses, and have been used as cancer vaccines in conjunction with an immunological adjuvant.^58^ However, CCNP preparations may be contaminated with nucleic acids derived from these cells, resulting in potential safety concerns about the carryover of such material, and thus cell membranes of non-malignant cell preparations are considered safer for use in exosome mimetics designed for clinical applications. Exosome-mimetic nanosystems (EMNs) that simulate cell-derived exosomes have been created using liposome technology, and demonstrate important advantages in production efficiency and functionality. EMN have important methodology and regulatory advantages, including faster preparation time than exosome isolates, with a 1000× production yield for EMNs versus exosomes, and greater drug loading efficiency for amphiphilic and hydrophobic compounds.^60^ In another study, artificial chimeric exosomes were constructed to display anti-phagocytosis and targeting functions by combining membrane proteins from red blood cells and tumor cells, resulting in exosome mimetics that exhibited low interception rates with higher tumor accumulation and better antitumor therapeutic effects than exosomes.^61^

Coating polymeric nanoparticles with cell membrane material represents an effective method for introducing multiple desirable membrane antigens and surface functionalities, which is difficult to achieve using traditional synthetic techniques. Preparation of such exosome-mimetics requires expertise with the isolation of cell membranes and particle functionalization, as well as special synthetic equipment, but coating polymeric nanoparticles with CCNPs presents an effective means to generate therapeutic agents for cancer immunotherapy and drug delivery, which can be customized for personalized cancer therapy.

**Figure 3:**
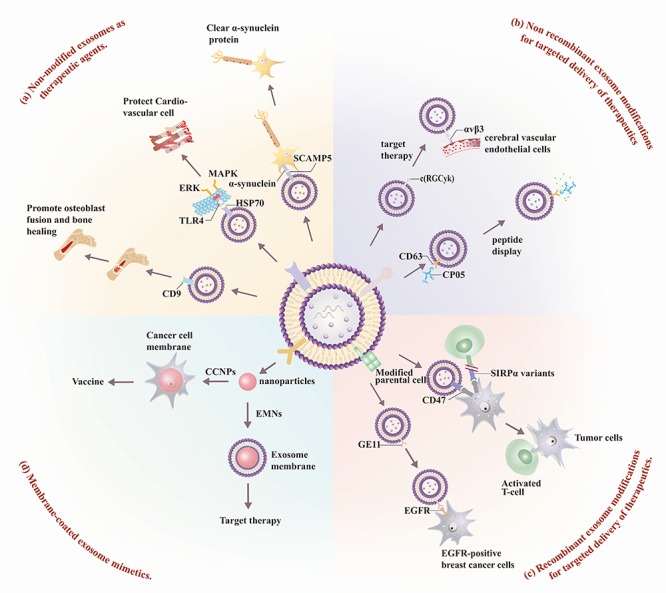
**Exosome membrane protein therapy**. Several approaches have been taken to develop exosome-based or exosome mimetic therapeutics. These include: **(a)** non-modified exosome therapeutics, employing native exosome surface proteins (SCAMP, HSP70, CD9) to recognize specific proteins to carry out specific tissue-directed functions; **(b)** non-recombinant exosome modifications; **(c)** recombinant exosome modifications; and (**d**) membrane-coated exosome mimetics.

Native or surface-modified exosomes, exosomes produced by genetically engineered cells, or exosome mimetics can be used for the targeted transport of drugs to diseased cells in various applications, each of which has its own advantages and disadvantages, as summarized in Fig. 3. Membrane proteins present on non-modified exosomes can have multiple beneficial therapeutic effects: CD9 expression can promote osteoblast fusion and bone healing^34^; 70 kDa heat shock protein (HSP70) can exert a cardio-protective effect by attenuating pro-inflammatory TLR4 signaling^47^^,^^49^; and SCAMP5 can promote α-synuclein clearance to attenuated neurotoxic α-synuclein aggregation associated with neurodegenerative disease.^51^ Simple surface modification approaches can alternately be employed to confer tissue specificity and/or therapeutic properties on isolated exosomes. Bioorthogonal conjugation has been employed to attach a c(RGDyK) peptide and confer specificity to integrin αvβ3 expressed in reactive cerebral vascular endothelial cells following ischemia for selective drug delivery, while the affinity of the CP05 peptide for CD63 has been used to regulate exosome functionalization and confer target specificity and drug delivery through their binding of drug- or ligand-modified CP05 peptides.^37^^,^^48^ Recombinant modification of cultured cells can also be employed to confer *de novo* membrane protein or peptide expression to alter the target specificity or functional activity of the engineered exosomes. This approach has been employed to produce exosomes that express SIRPα and block CD47-SIRPα interactions between tumor cells and T lymphocytes,^55^ and to generate exosomes expressing the EGFR-specific peptide GE11 to promote their interaction with EGFR-positive breast cancer cells.^57^ Finally, exosome mimetics coated with CCNPs or EMNs to confer the biocompatibility and/or targeting properties of these membranes with desired characteristics of nanoparticles in cancer vaccine or targeted therapy approaches, while reducing the variability and labor involved with other exosome-based approaches.^58^^,^^60^ This array of options allows researchers to select an exosome therapeutic approach based on their experimental needs and limitations.

## Perspective

All cells appear to secrete exosomes, and exosomes are present in all body fluids. Exosomes carry biological and genetic information that can identify their parental cell types, and can transfer their native or engineered contents to specific recipient cell types through various interaction mechanisms to influence their phenotypes and fates. These properties have led to research on the development of exosome biomarkers of disease and therapeutic strategies that employ the properties of native or modified exosomes or exosome mimetics. Most current exosome biomarker research focuses on serum/plasma exosome analysis for correlations with tumor phenotypes, but there is also potential for analysis of exosomes in other tissues to advance the diagnosis of specific tumor types (e.g. saliva and oral tumors, urine and urinary tract tumors, etc.).

Proteomics and living cell imaging have led to the discovery that exosome membrane proteins play key roles in exosome-mediated information transfer. Characterization of disease-specific exosome membrane proteins and better understanding of the physio-pathologic roles of these proteins in their respective diseases has significant implications for the development of future clinical applications using this information for improved diagnostics or therapeutics.

Exosomes have advantages over many other therapeutic platforms (e.g. low immunogenicity, specific organotropism, and inherent fusogenic activities) for the delivery of membrane proteins or drug cargoes. However, low yield rates of both native and modified or engineered exosomes, and concern over the safety and off-target effects of their additional components serve to limit their path to clinical applications. Direct or indirect modification of exosome membrane proteins can expand the function and application scope of exosome therapeutics, but further increase concerns about the purification, characterization, and safety of the resulting exosome therapeutics. Recent research has appeared to focus on the development of nanoparticle-based exosome mimetics that can be precisely engineered to display desired characteristics; however, additional validation studies are required to demonstrate that exosome-based therapeutics are suitable for clinical applications.

## Author contributions

Q.H and H.S contribute equally to this work. Conceptualization, M.H.W. and T.Y.H.; writing—original draft preparation, Q.H; writing—review and editing, C.L. and W.F.T.; visualization, H.S. and J.L.; supervision, W.F.T. and T.Y.H.; All the authors read the article and approved the final version.
